# Effect of textile dyes on activity and differential regulation of laccase genes from *Pleurotus ostreatus* grown in submerged fermentation

**DOI:** 10.1186/s13568-016-0263-3

**Published:** 2016-10-07

**Authors:** Verónica Garrido-Bazán, Maura Téllez-Téllez, Alfredo Herrera-Estrella, Gerardo Díaz-Godínez, Soley Nava-Galicia, Miguel Ángel Villalobos-López, Analilia Arroyo-Becerra, Martha Bibbins-Martínez

**Affiliations:** 1 Centro de Investigación en Biotecnología Aplicada-Instituto Politécnico Nacional, Carretera Estatal Sta Inés Tecuexcomac-Tepetitla, km. 1.5, C.P: 90700 Tepetitla de Lárdizabal, Tlaxcala Mexico; 2 Centro de Investigaciones Biológicas, Universidad Autónoma del Estado de Morelos, Cuernavaca, Morelos Mexico; 3 Laboratorio Nacional de Genómica para la Biodiversidad, Cinvestav, Irapuato, Gto Mexico; 4 Laboratory of Biotechnology, Research Center for Biological Sciences, Universidad Autónoma de Tlaxcala, Tlaxcala, Mexico

**Keywords:** Laccases, Isoenzymes, *Pleurotus ostreatus*, Gene expression, RT-qPCR

## Abstract

This research was conducted to extend the knowledge on the differential regulation of laccase genes in response to dyes. In order to accomplish this, we analyzed both, the expression of five laccase genes by real time RT-qPCR, and also the laccase activity and isoforms patterns during the time-course of a *Pleurotus ostreatus* submerged fermentation supplemented with either acetyl yellow G (AYG) or remazol brilliant blue R (RBBR) dyes. For the purpose of obtaining a stable reference gene for optimal normalization of RT-quantitative PCR gene expression assays, we tested four candidate reference genes. As a result of this analysis, *gpd* was selected as reference index for data normalization. The addition of dyes had an induction effect on the enzymatic activity and also modified the zymogram profile. Fermentation with RBBR showed the highest laccase activity and number of isoforms along the course of the fermentation. Laccase gene expression profiles displayed up/down regulation along the fermentation time in four laccase genes (*pox4*, *pox3*, *poxa1b* and *pox2*), while *pox1* was not expressed in either of the fermentation conditions. AYG addition caused the highest induction and repression levels for genes *pox3* and *poxa1b* respectively. The expression level for all genes in the presence of RBBR were lower than in AYG, being in both conditions this response growth time dependent. These results show the influence of the nature of dyes on the induction level of laccase activity and on the differential regulation of the laccase genes expression in *P. ostreatus*.

**Electronic supplementary material:**

The online version of this article (doi:10.1186/s13568-016-0263-3) contains supplementary material, which is available to authorized users.

## Introduction

Of all industrial sector effluent, wastewater from the textile industry is classified as one of the most polluting, in terms of both volume and composition (Vandevivere et al. 1998; López et al. 2006). Inefficient industrial textile processes produce residual water with a high concentration of synthetic dyes (Asgher et al. 2009). Currently, more than 10,000 different dyes and pigments are used in the dyeing and printing industry worldwide. World production has been estimated at 800,000 tons per year, with at least 10–15 % of the pigments used discharged into the environment through wastewater (Levin et al. 2004; Palmieri et al. 2005; Revankar and Lele 2007). Many textile dyes are believed to be toxic or carcinogenic (Hamedaani et al. 2007). These compounds are considered xenobiotics and to be recalcitrant, and, in most cases, are very difficult to remove.

Due to fungal peroxidases comprising mainly laccases, manganese peroxidases, lignin peroxidases, and veratryl alcohol oxidases (Wesenberg et al. 2003; Swamy and Ramsay 1999; Tavčar et al. 2006), white rot fungi are organisms capable of degrading a variety of compounds, including textile dyes (López et al. 2006).

Laccases (benzenediol: oxygen oxidoreductases, EC 1.10.3.2) are glycoproteins classified as multi-copper oxidases that use the distinctive redox ability of copper ions to concomitantly catalyze the oxidation of a wide range of aromatic substrates with the reduction of molecular oxygen to water (Thurston 1994; Solomon et al. 1996). Given their high and non-specific oxidation potential laccases are biocatalysts useful for a wide range of biotechnology applications. These enzymes are used efficiently in the detoxification of the wastewater produced in pulp bleaching processes (Bajpai 1999), in the treatment of wastewater from industrial plants (Durán and Esposito 2000), the enzymatic modification of fibers and the decoloration of effluent (Abadulla et al. 2000).

*Pleurotusostreatus* has been reported to contain several laccase isoenzymes encoded by multigene families (Giardina et al. 2010). These isoenzymes often present differences in terms of their catalytic properties, regulation mechanisms and location. The transcriptional activity of laccase encoding genes is often regulated by metal ions (Collins and Dobson 1997; Galhaup et al. 2002), aromatic compounds or lignin derivatives (Terrón et al. 2004), as well as the source and concentration of nitrogen (Collins and Dobson 1997) and/or carbon (Soden and Dobson 2001). The above mentioned factors may act synergistically or antagonistically (Baldrian and Gabriel 2002; Faraco et al. 2003; Periasamy and Palvannan 2010).

The physiological mechanisms that control fungal development are also known to modulate the expression levels of laccase isoenzymes, since some isoenzymes have been observed during the exponential growth phase, and could participate in the degradation of the substrate. Other isoenzymes have been found during the stationary phase, which may be related to both morphogenesis processes and spore pigmentation (Temp and Eggert 1999; Lettera et al. 2010). Several reports indicate that laccases produced by *P. ostreatus* are the main enzymes that mediate dye decolourisation, due to their enzymatic properties and also their potential for degrading dyes of diverse chemical structure, therefore the development of processes based on laccases represent an effective tool for application in the textile effluent degradation (Palmieri et al. 2005).

The main objective of this research was to study the effect of chemically different dyes on the production and the differential regulation of laccase genes from *P. ostreatus*.

## Materials and methods

### Organism

A strain of *P. ostreatus* from the American Type Culture Collection (ATCC 32783) (Manassas, Virginia, USA) was used.

### Submerged cultures

The fermentations were performed in 125 mL Erlenmeyer flasks containing 50 mL of basal medium (BM) of the following composition (g/L): yeast extract, 5; glucose, 10; K_2_HPO_4_, 0.4; ZnSO_4_·7H_2_O, 0.001; KH_2_PO_4_, 0.6; FeSO_4_·7H_2_O, 0.05; MnSO_4_·H_2_O, 0.05; MgSO_4_·7H_2_O, 0.5; CuSO_4_·7H_2_O, 0.25 (Téllez-Téllez et al. 2008). Three fermentations of *P. ostreatus* grown in basal medium (BMF) and in the presence of either 500 ppm of RBBR (remazol brilliant blue R dye, SIGMA) (BBF) or 500 ppm of AYG (acetyl yellow G, ALDRICH) (AYF) were established. Each flask was inoculated with three mycelial plugs taken from the periphery of *P. ostreatus* colonies grown for 7 days at 25 °C in Petri dishes containing potato dextrose agar. The cultures were incubated at 25 °C for 23 days on a rotary shaker at 120 rpm. Three flasks were taken as samples at 120, 168, 240, 288, 336, 408, 480 and 576 h of fermentation. The enzymatic extract (EE) was obtained by filtration of the cultures using filter paper (Whatman No. 4), and stored at −20 °C until it was analyzed, while the mycelium was rinsed with 0.9 % NaCl and stored at −70 °C until the total RNA extraction procedure was conducted or used for biomass (X) determination as difference of dry weight (g/L) (Additional file 1: Figure S1). Experiments were performed in triplicate, with the values shown being representative of at least two of the experiments.

### Enzyme assays

Laccase activity was determined by measuring changes in absorbance at 468 nm with extinction coefficient ɛ_468_ = 35,645 M^−1^cm^−1^, using 2,6-dimethoxyphenol (DMP) as the substrate. The assay mixture contained 950 μl of substrate (2 mM DMP in 0.1 M phosphate buffer at pH 6.5) and 50 μl EE, and was incubated at 40 °C for 1 min (Téllez-Téllez et al. 2008). The activity was expressed in international units (U/mL).

### Zymogram analysis

Laccase activity was also detected through zymograms, using the modified SDS-PAGE technique (Laemmli 1970). The running gel contained 100 g acrylamide/L and 27 g bis-acrylamide/L. The stacking gel contained 40 g acrylamide/L and 27 g bis-acrylamide/L. Each EE (20 µl approx.) was mixed with sample buffer without a reducing agent for the disulphide bonds. The samples were placed in Mini-Protean III electrophoresis system (BioRad) gels (thickness 0.75 mm) with 150 V then applied for 1–1.25 h. After the electrophoresis, the gels were washed with deionized water on an orbital shaker (20–30 rpm) for 30 min, with the water changed every 10 min to remove SDS. Finally, the gels were incubated at room temperature in substrate solutions (2 mM DMP). Laccase activity bands from the oxidation of the substrate appeared on the gel after approximately 1 h (Téllez-Téllez et al. 2008).

### Nucleic acid extraction and real time qPCR

Total RNA was isolated from frozen mycelia harvested at different fermentation times, using TRIZOL (Invitrogen) extraction, and was spectrophotometrically quantified by determining the absorbance ratio at OD260/280. RNA was treated with RNAse-free DNase I (Invitrogen). The final RNA concentration was set to 500 ng/µl. Subsequently, 1 µg of total RNA was reverse-transcribed into cDNA in a 20 µl volume using the SuperScript^™^II Reverse Transcriptase (Invitrogen) by following the manufacturer protocol.

The procedure for reverse transcription quantitative PCR experiments was adapted from (Castanera et al. 2015). RT-qPCRs were performed in a StepOnePlus® (Applied Biosystems), using SYBR green dye to detect product amplification. A set of specific primers was designed for the amplification of the transcript from the four laccase genes identified in the genome (Table 1). Primers corresponding to the panel of reference genes were designed using the filtered model transcript sequence of PC15 (v2.0) (http://www.jgi.doe.gov) and the Express Primer Express^®^ 3.0 (Applied Biosystems) (Additional file 1: Table S1). With a final volume of 20 µl, each reaction mixture contained 10 µl Maxima Probe/ROX qPCR Master Mix (2X) (ThermoScientific), 200 nM forward and reverse primer, and a 1 µl 1:10 dilution of the RT product. Amplifications were performed with an initial 5 min step of 95 °C followed by 40 denaturation cycles at 95 °C for 30 s and primer annealing and extension at 60 °C for 40 s. The melting curves ranged from 60 to 95 °C and temperature was increased in increments of 0.3 °C. StepOne software was used to confirm the occurrence of specific amplification peaks. All RT-qPCR reaction were carried out in triplicate with template-free negative control being performed in parallel. The crossing-point (Cp) values and relative fluorescence units were recorded, with the latter used to calculate amplification efficiencies via linear regression. The PCR efficiency (E) and the regression coefficient (*R*2) were calculated using the slope of the standard curve according to the equation E = [10−(1/slope)−1] × 100 %.

**Table 1:** Primer sequence, product length and amplification efficiencies used in this study

Gene	Transcript ID^a^	Orientation^b^	Sequence (5′–3′)	Product size (bp)	Efficiency (%)
*poxa1b*	1113032	Fw	GGCGACAGGTTCCAAATTA	101	2.23
		Rv	TTGTGTCCCTTGACGAAGAG		
*pox2*	1089723	Fw	CTGGCGTTCTCGTTCAAG	87	2.12
		Rv	TCGTCTTCAACATAGTCGTGTCT		
*pox3*	1077328	Fw	TCACCATTCGCTTTGTCACT	100	2.14
		Rv	TTCTCAGCCAATACGACAGC		
*pox4*	1043420	Fw	TACTCGTTCGTGTTGAAGGC	131	2.27
		Rv	GCATTGGGTGCTAGGATGTA		
*gpd*	1090672	Fw	GCTGACGCACCAATGTTC	83	2.00
		Rv	GTGCAAGACGCATTTGAG		

^a^Transcript ID and gene nomenclature refer to the annotation of *P. ostreatus* PC15 genome version 2.0 (http://www.genome.jgi-psf.org/PleosPC15_2/PleosPC15_2. home.html)

^b^ *Fw* forward; *Rv* reverse

### Reference genes, quantification of RT-qPCR data, and statistical analyses

Four genes of different functional class were selected as reference candidates. The gene panel used in this study contained housekeeping genes, such as glyceraldehyde 3-phosphate dehydrogenase *(gpd)*, β-tubulin (*tub)*, actin *(act)* and peptidase *(pep)* (Additional file 1: Table S1). The expression of the genes was evaluated in six samples corresponding to our experimental conditions. GeNorm (Vandesompele et al. 2002) and NormFinder (Andersen et al. 2004) algorithms were applied to rank the four candidates according to their expression stability, and a reference index consisting of the geometric mean of the best-performing candidates was used for RT-qPCR data normalization.

Data pre-processing was performed using Microsoft Excel 2007 and included efficiencies and reference gene normalization. The fold expression was calculated by the 2^−Δ ΔCt^ method as described by (Pfaffl 2001) (Eq. 1).

1 $$\text{ratio =}\frac{(E_{\text{target}})^{\Delta{\text{Cp}}_{\text{target}}(\text{control}-\text{sample})}}{(E_{\text{ref}})^{\Delta{\text{Cp}}_{\text{ref}}(\text{control}-\text{sample})}}$$

In the above equation E_target_ is the real-time PCR efficiency of target gene transcript: E_ref_ is the real-time PCR efficiency of a reference gene transcript; ΔCp_target_ is the CP deviation of control-sample of the target gene transcript. All other multiples comparisons were performed using the statistical analysis software SAS 2002 by SAS Institute Inc., Cary, NC, USA.

## Results

### Effect of dyes on laccase activity

**Fig. 1:**
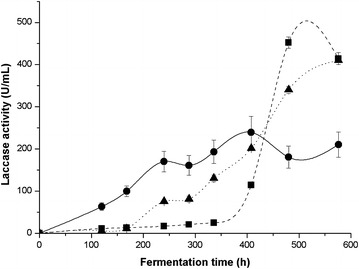
Time course of extracellular laccase activity of *P. ostreatus* obtained in submerged fermentations in BMF (■ *blacksquare *), BBF (● *black circle*) and AYF (▲ *black triangle*) media. The *error bars* represent the standard deviation of three different fermentation runs

*Pleurotus ostreatus* was grown in liquid fermentation at 25 °C for 23 days. Samples were taken at regular intervals and filtrated, with the supernatant obtained then used to measure laccase activity. Figure 1 shows the laccase activity, which increased from the beginning of fermentation in BMF, with maximal activity observed at 408 h (239 U/mL). In BBF, the activity was low from the beginning of the fermentation until 336 h (approx. 25 U/mL), after which the activity increased and reached its peak at 480 h (452 U/mL), while, in AYF, the activity was low until 168 h of fermentation (approx. 11 U/mL) with the maximal activity value being 410 U/mL at 576 h.

### Effect of dyes on laccase isoenzymes production

**Fig. 2:**
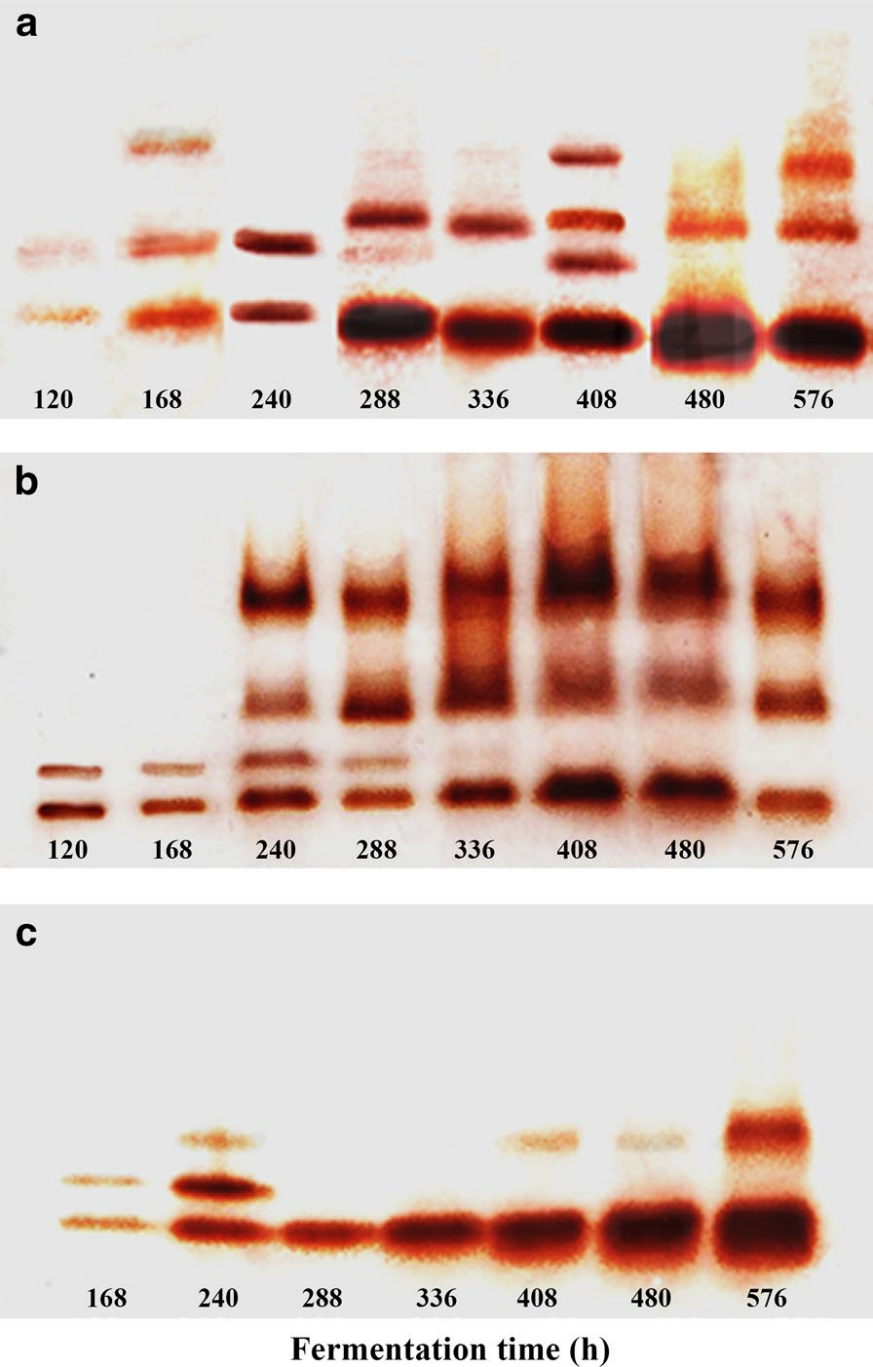
Zymograms of laccase isoenzymes produced by *P. ostreatus* grown in basal medium BMF (**a**) and in the presence of either 500 ppm of remazol brilliant blue R dye BBF (**b**) or 500 ppm of acetyl yellow G dye AYF (**c**)

Laccase isoenzymes produced during the fermentation process are shown in Fig. 2. Two to four isoenzymes were observed in enzymatic extracts (EEs) obtained from the BMF (Fig. 2a). Figure 2b shows the laccase isoenzyme profile obtained in BBF, in which two isoenzymes were observed in EE collected at 120 and 168 h, four isoenzymes at 240 and 288 h and three at the later stages of the fermentation.

The growth of the fungus in AYF resulted in EEs with less isoenzymes than BMF and BBF, with one isoenzyme observed at 288 and 336 h of fermentation, two isoenzymes observed at 168, 408, 480 and 576 h, and only three isoenzymes observed at 240 h (Fig. 2c).

### Identification and validation of reference genes for qPCR analysis

To evaluate the stability of the reference genes across experimental conditions, the transcript abundance of the four candidate reference genes were detected by their mean Ct values (Additional file 1: Figure S2). The GeNorm algorithm identified *gpd* and *act* as the most stable genes along all the conditions assayed, displaying an expression stability value (M-value) of 0.213. In addition NormFinder algorithm identifier *gpd* as most stable gene (Additional file 1: Figure S3). As a consequence of this analysis, *gpd* was selected as reference index for data normalization.

### Effect of dyes on the expression of laccase genes

**Fig. 3:**
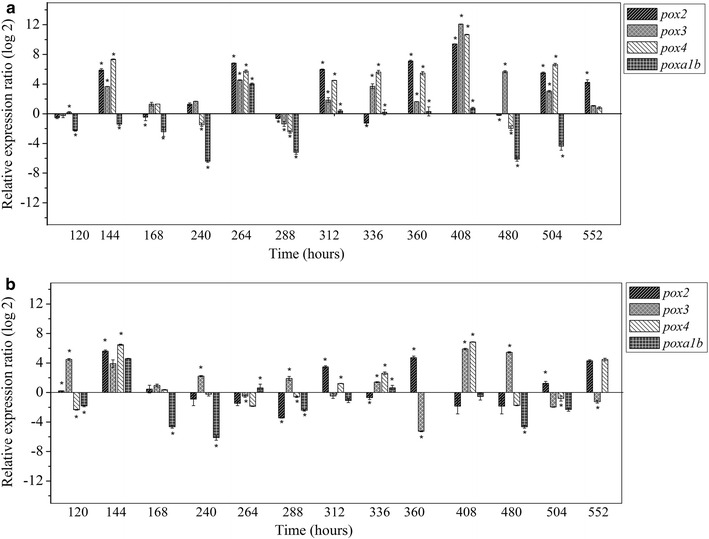
Expression levels of four laccase genes from *P. ostreatus* during time course fermentation with the addition of acetyl yellow G dye (**a**) and remazol brilliant blue R dye (**b**). *Error bars* represent the standard deviations of the means of three independent amplifications, and *asterisks* mean that the changes referred to the basal condition are statistically significant at p < 0.05

The expression of laccase genes *pox1*, *pox2, pox3, pox4* and *poxa1b* in response to the addition of dyes was evaluated at transcriptional level. First *P. ostreatus* was grown in BMF (reference condition). Then we monitored by RT-qPCR using specific primers the time course (120–552 h) of transcriptional changes of the five laccase genes in both fermentations supplemented with dyes (BBF and AYF). The 2^−ΔΔCt^ method (Pfaffl 2001) was applied to the transcriptional analysis to quantify the relative expression of each gene with respect to the corresponding un-induced value for the given time point (reference condition). Figure 3a, b shows the laccase gene expression profiles where, in general terms, RBBR and AYG dyes display up/down regulation along the fermentation time in four laccase genes (*pox4*, *pox3*, *poxa1b* and *pox2*), while *pox1* was not expressed in any of the of the two fermentation conditions. AYG addition caused the highest induction in the transcript level of gene *pox3* that becomes several order of magnitude higher than that of the other analyzed genes (up to 12-fold increase) followed by *pox4* (tenfold increase) and *pox2* (ninefold increase) all of them at 408 h (Fig. 3a). On the other hand *poxa1b* showed the highest down regulation (−6.39-fold) at 240 h and remains almost constant along the fermentation time. The expression level for all genes in the presence of RBBR (Fig. 3b) were lower than in AYG, *pox4* showed the highest induction (6.85- and 6.47-fold) at 408 and 144 h respectively, followed by *pox3* (5.89-fold) at 408 h and *pox2* (5.61-fold), *poxa1b* (4.60-fold) both of them at 144 h.

### Percentage contribution of each pox gene to the global laccase expression

**Fig. 4:**
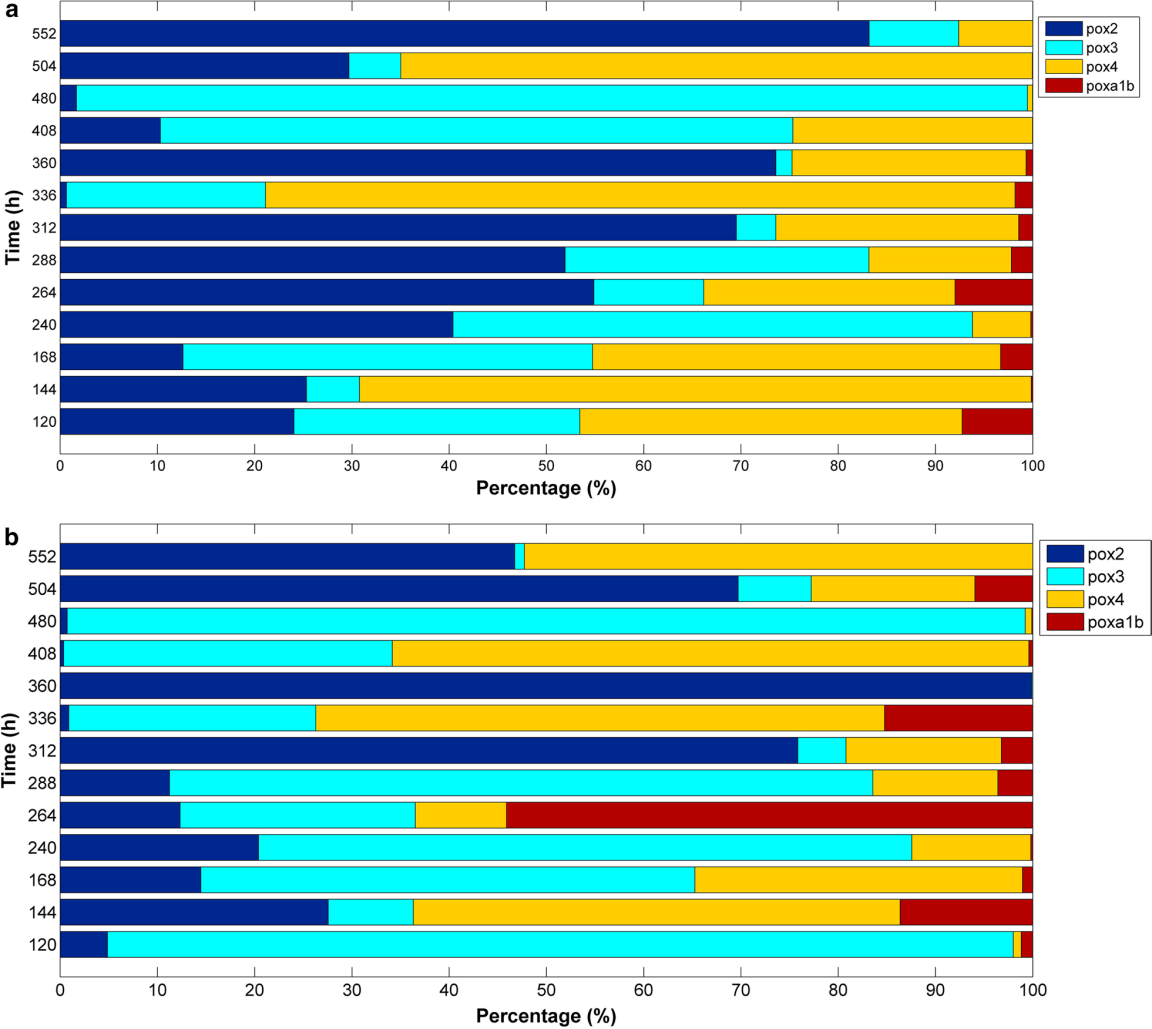
Relative expression of laccase genes as percentage of the total expression in AYF (**a**) and BBF (**b**)

In order to analyze the contribution of each *pox* gene to the total relative expression on the time-course fermentation, their transcriptional levels were also shown as percentage of the total expression (Fig. 4). For AYG fermentation, *pox2* represents 36.7 % of the total laccase expression followed by *pox4* (32.31 %), *pox3* (28.96 %) and *poxa1b* (1.9 %) (Fig. 4a) on the other hand for RBBR fermentation the contribution was *pox3* (37.5 %), *pox2* (29.46 %), *pox4* (25.22 %) and *poxa1b* (7.59 %) (Fig. 4b). It is clear that changing the type of dye in the fermentation lead to different transcriptional profiles for each laccase gene with up and down regulation depending on the fermentation sampling time. Furthermore the addition of dyes to the culture medium caused a strong induction of *pox3* and *pox4* and to a lesser extent to *pox2* and *poxa1b* being this response growth time dependent. On the other hand, the transcriptional level of genes *pox2*, *pox3* and *pox4* represent the main contribution to the global laccase expression.

## Discussion

The highest laccase activity was produced in the stationary growth phase of the fungi in all conditions applied in this study. However the addition of either Remazol brilliant blue R or acetyl yellow G dyes had an induction effect on the enzymatic activity, which almost doubled for both dyes in comparison with basal fermentation. The addition of phenolic and aromatic compounds, such as the dyes used in this study, has been proven to increase laccase production, given that laccase induction by phenolic substances is a putative response mechanism developed by fungi against toxic compounds (Pezzella et al. 2013; Casas et al. 2013). On the other hand, the induction level mediated by dyes has been reported to be highly sensitive to small differences in their chemical structures (Vanhulle et al. 2007). In this work we used azo (RBBR) and sulphonic (AYG) dyes and the differential effect was observed on both, laccase activity and gene expression level.

The appearance of laccase gene families is very common in fungi, with the synthesis and secretion of each family member strongly influenced by nutrient level, culture conditions, and developmental stage. While the genome of *P. ostreatus* contains 11 laccase encoding genes, to date only six laccase isoenzymes have been isolated and characterized. (Pezzella et al. 2013): POX2 (59 kD SDS-PAGE) (Palmieri et al. 1993), POXA1w (57 kDa) (Palmieri et al. 1997), POXA1b (62 kDa) (Giardina et al. 1999), POXA2 (61 kDa) (Palmieri et al. 1997), POXA3a and POXA3b (67 kDa) (Palmieri et al. 2003). POX2 is a typical laccase and is the most widely produced under different growth conditions (Palmieri et al. 1993). POXA1b is a neutral blue laccase, very stable at alkaline pH (Giardina et al. 1999) and with a high redox potential (Garzillo et al. 2001). Other laccase encoding genes have been identified in *P. ostreatus* such as *pox3*, *pox4* and *pox5*, though their corresponding proteins have never been isolated in culture broth. The heterologous expression of these genes in the yeasts *S. cerevisiae* and *K. lactis* produced very unstable laccases with expression problems (Pezzella et al. 2009).

The profile of laccase isoenzymes can be the result of either the expression of different genes or postransductional modifications. As shown in Fig. 2, zymograms taken during this research showed up to four isoenzymes; however, the addition of dyes also modified the zymographic pattern, with the condition with the highest laccase activity and highest number of isoforms during the fermentation being the fermentation conducted in the presence of the remazol brilliant blue R dye. It has been reported that *P. ostreatus* grown on agar with starch as a carbon source presented two isoenzymes at an initial pH of 6.5 (Téllez-Téllez et al. 2005). Téllez-Téllez et al. (2008) grew *P. ostreatus* in submerged fermentation at pH 6.5 and observed two and four isoenzymes during the exponential and stationary growth phases, respectively. Recently, the number of *P. ostreatus* laccase isoenzymes in buffered and non-buffered media was determined with the initial pH adjusted to 3.5 in both culture media. One laccase isoenzyme was produced in both media during the entire fermentation process. In the non-buffered medium, an additional isoenzyme of lower molecular weight than that produced in the entire fermentation process was produced at the beginning of the exponential phase of growth when the pH reached a value of 6.5 (Díaz et al. 2011).

Laccase gene transcription is regulated by metal ions, various aromatic compounds related to lignin or lignin derivatives, nitrogen and carbon sources, factors which cause specific laccase transcriptional profiles with variations among not only different species but also different isoforms in the same strain (Piscitelli et al. 2011; Pezzella et al. 2013). As expected, the transcriptional profiles differ in this study depending on the condition tested. However, the addition of dyes resulted in the induction of all genes evaluated except gene *pox1*, an effect which was observed from the beginning of the fermentation onwards.

In the case of gene *pox1*, there was no amplification in any fermentation, with some reports indicating that the laccase isoenzyme gene *pox1* is closely related to *pox2*, since their cDNA sequences show 84 % similarity (Giardina et al. 1995). Pezzella et al. (2013) reported that, due to the high sequence similarity between *pox1* and *pox2*, it is difficult to distinguish their expression profiles in *P. ostreatus*. This means that transcription levels are considered as the sum of both genes (*pox1*/*pox2*); however, a more comprehensive analysis would be needed in order to arrive at this conclusion in this study. On the other hand, while *pox2* was amplified in all conditions evaluated in this study, the dyes induced its expression. It has been reported that the promoter of *pox2* contains at least eight putative metal-responsive elements (MRE), which leads to a strong transcriptional induction being observed in the copper-supplemented culture (Moussa 2009; Amore et al. 2012). Given that the basal media used in this research was also supplemented with copper, this might explain the transcriptional profile obtained in the basal fermentation for gene *pox2*. Furthermore, the promoter region of the *pox2* gene also shows a possible xenobiotic responsive element (XRE), with industrial dyes being considered xenobiotics. Pezzella et al. (2013) reported that *pox*2 may fulfill this role during vegetative growth, which might explain why expression was observed during the complete fermentation process. In addition, POX2 has been reported to be the enzyme most abundantly produced under several growth conditions (Palmieri et al. 2005; Castanera et al. 2012; Parenti et al. 2013). The most marked effect of dyes in the transcription induction were for *pox3* and *pox4* genes, where *pox3* presented the highest induction level up to 12-fold increase. Interestingly the promoter region of *pox3* presented three putative XREs compare with just one for *pox2* and none for *pox4* and *poxa1b*. In addition nucleotide sequence analysis predicted the presence of 5 and 1 MRE in *pox3* and *pox4* respectively (Pezzella et al. 2009). This results may suggests a dye-responsive induction pathway. However location and orientation of such and other responsive elements may also play a role in dye response. The close relationship between *pox2*, *pox1* and *pox4* genes has been reported, where they present exactly the same gene organization, while, on the contrary, *pox3* exhibits a very different structure from that of the other family members (Janusz et al. 2013; Pezzella et al. 2009). *poxa1b* showed the highest repression level of all genes evaluated, in both fermentations conducted with dyes added and seems to be the most affected by copper and/or dye among the *P. otreatus* laccase transcripts analyzed in this research, with this response possibly being growth time dependent. Our results are in agreement with Pezzella et al. (2013) who reported that *poxa1b* (*lacc6*) was barely induced in the presence of two inducers (Cu-ferulic acid) and its induction was limited to the latest stage of cultivation (7th day). Analysis of *poxa1b* promoter showed the presence of several putative responsive elements, such as antioxidant response element (ARE) and MREs but not XREs, C and N nutrient responsive elements (Amore et al. 2012; Miele et al. 2010; Piscitelli et al. 2011), the lack of XREs may explain the barely induction level observed for this gene under the assays conditions evaluated in this study.

The activity, isoforms and transcriptional profiles obtained in this investigation show the complex regulation of the laccase genes by xenobiotic compounds such as the dyes tested in combination with such other factors as culture conditions, developmental stage, and variations in medium composition during *P. ostreatus* growth.

The textile dyes RBBR and AYG acted as inducers of laccase activity and modified the zymographic and expression profiles of laccase genes. Laccase activity may be defined by the expression of genes *pox2*, *pox3* and *pox4* and the oxidation of the dyes under study may be the result of this gene products. The high induction level of genes *pox3* and *pox4* mediated by dyes suggests that the laccase coded by them could by the main activity present in the dye fermentations. Given what is known about the presence of response elements (metal ions, xenobiotics, stress, glucose, and nitrogen) in laccase gene promoters, the dyes may be involved in the regulation of expression. However, the precise molecular mechanism that regulates gene expression through these potential response elements is unknown and needs to be fully explored in future work.

## Additional file

10.1186/s13568-016-0263-3 Growth of *P. ostreatus* and pH profile in submerged fermentations in BMF (●black circle), BBF (■ black square) and AYF (♦black diamond) media. The error bars represent the standard deviation of three different fermentation runs. **Figure S2.** Genorm analysis of the expression stability of 4 reference genes. **Figure S3.** Variability of Cp values of 4 reference genes tested under the 3 different fermentation conditions using NormFinder. **Table S1.** Identifiers and product lengths of reference genes primers used in this study.

## Abbreviations

AYG: acetyl yellow G
RBBR: remazol brilliant blue R
BM: basal medium
BMF: basal medium fermentation
BBF: remazol brillliant blue R fermentation
AYF: acetyl-yellow G fermentation
EE: enzymatic extract
DMP: 2,6-dimethoxyphenol
RT: qPCR- reverse transcription-quantitative PCR
gpd: glyceraldehyde 3-phosphate dehydrogenase
tub: β-Tubulin
pep: peptidase
