# Supplementary material for: Effect of textile dyes on activity and differential regulation of laccase genes from Pleurotus ostreatus grown in submerged fermentation
Source: AMB Express. 2016 Oct 7;6:93. doi: 10.1186/s13568-016-0263-3 (PMC5055507; doi:10.1186/s13568-016-0263-3)

**AMB Express JOURNAL**

**Effect of textile dyes on activity and differential regulation of laccase genes from *Pleurotus ostreatus* grown in submerged fermentation**

Garrido-Bazán V^1^, Téllez-Téllez M^2^, Herrera-Estrella A^3^, Díaz-Godínez G^4^, Nava-Galicia SB^1^, Villalobos-López MA^1^, Arroyo-Becerra A^1^, and Bibbins-Martínez MD^1*^

^1^Centro de Investigación en Biotecnología Aplicada–Instituto Politécnico Nacional, Tlaxcala, México.

^2^Centro de Investigaciones Biológicas, Universidad Autónoma del Estado de Morelos, Morelos, México

^3^Laboratorio Nacional de Genómica para la Biodiversidad. Cinvestav-Irapuato, Gto,México

^4^Laboratory of Biotechnology, Research Center for Biological Sciences, Universidad Autónoma de Tlaxcala, Tlaxcala, México.

*CORRESPONDING AUTOR: Dr. Matha Bibbins-Martínez. Centro de Investigación en Biotecnología Aplicada –Instituto Politécnico Nacional. Carretera Estatal Sta Inés Tecuexcomac-Tepetitla, km. 1.5. Tepetitla de Lárdizabal, Tlaxcala, México. C.P: 90700.

e-mail: [mbibbinsm@ipn.mx/](mailto:mbibbinsm@ipn.mx/) [marthadbm1104@yahoo.com.mx](mailto:marthadbm1104@yahoo.com.mx)

tel/fax: +52555-7296000 ext. 87822/+522484870765

**Table S1** | Identifiers and product lengths of reference genes primers used in this study

| Gen | Transcript ID^a^ | Orientatio^b^ | Sequence (5’- 3’) | Product size (bp) |
| --- | --- | --- | --- | --- |
| *gpd* | 1090672 | Fw | GCTGACGCACCAATGTTC | 83 |
|  |  | Rv | GTGCAAGACGCATTTGAG |  |
| *act* | 25490 | Fw | CCTCTTCTGCTCCGTTCAA | 149 |
|  |  | Rv | CAATATCAATCCGCCGTATG |  |
| *β-tub* | [1076198](http://genome.jgi.doe.gov/cgi-bin/dispGeneModel?db=PleosPC15_2&id=1076198) | Fw | CGGTTCTGACTACTCACACGA | 134 |
|  |  | Rv | AATAAGGCGGTTCAAGTTGG |  |
| *pep* | 1092697 | Fw | CGGAGGACATTCTTGTTCAC | 142 |
|  |  | Rv | AGATCGGTAACCCACACGAG |  |

**Note**: ^a^Transcript ID and gene nomenclature refer to the annotation of *P. ostreatus* PC15 genome version 2.0 (http://genome.jgi-psf.org/ PleosPC15_2/PleosPC15_2. home.html).^b^Fw, Forward; Rv, reverse

**Supplementary figure legends**

**Figure S1.** Growth of *P. ostreatus* and pH profile in submerged fermentations in BMF (●black circle), BBF (■ black square) and AYF (♦black diamond) media. The error bars represent the standard deviation of three different fermentation runs

**Figure S2**. Genorm analysis of the expression stability of 4 reference genes

**Figure S3**. Variability of Cp values of 4 reference genes tested under the 3 different fermentation conditions using NormFinder


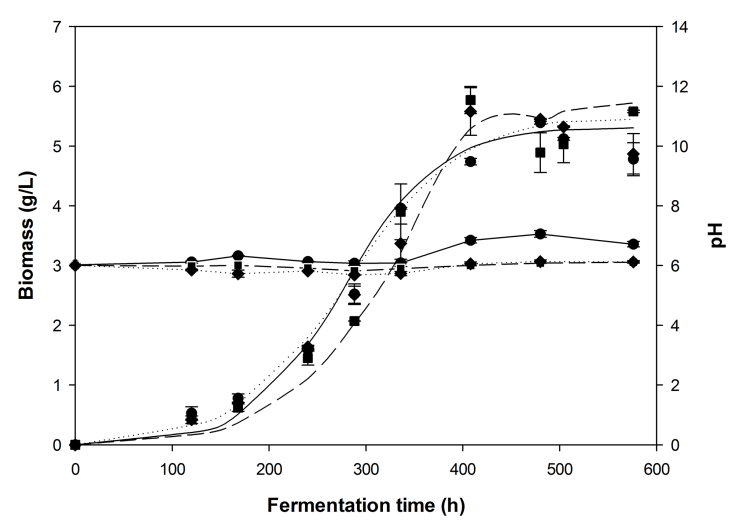


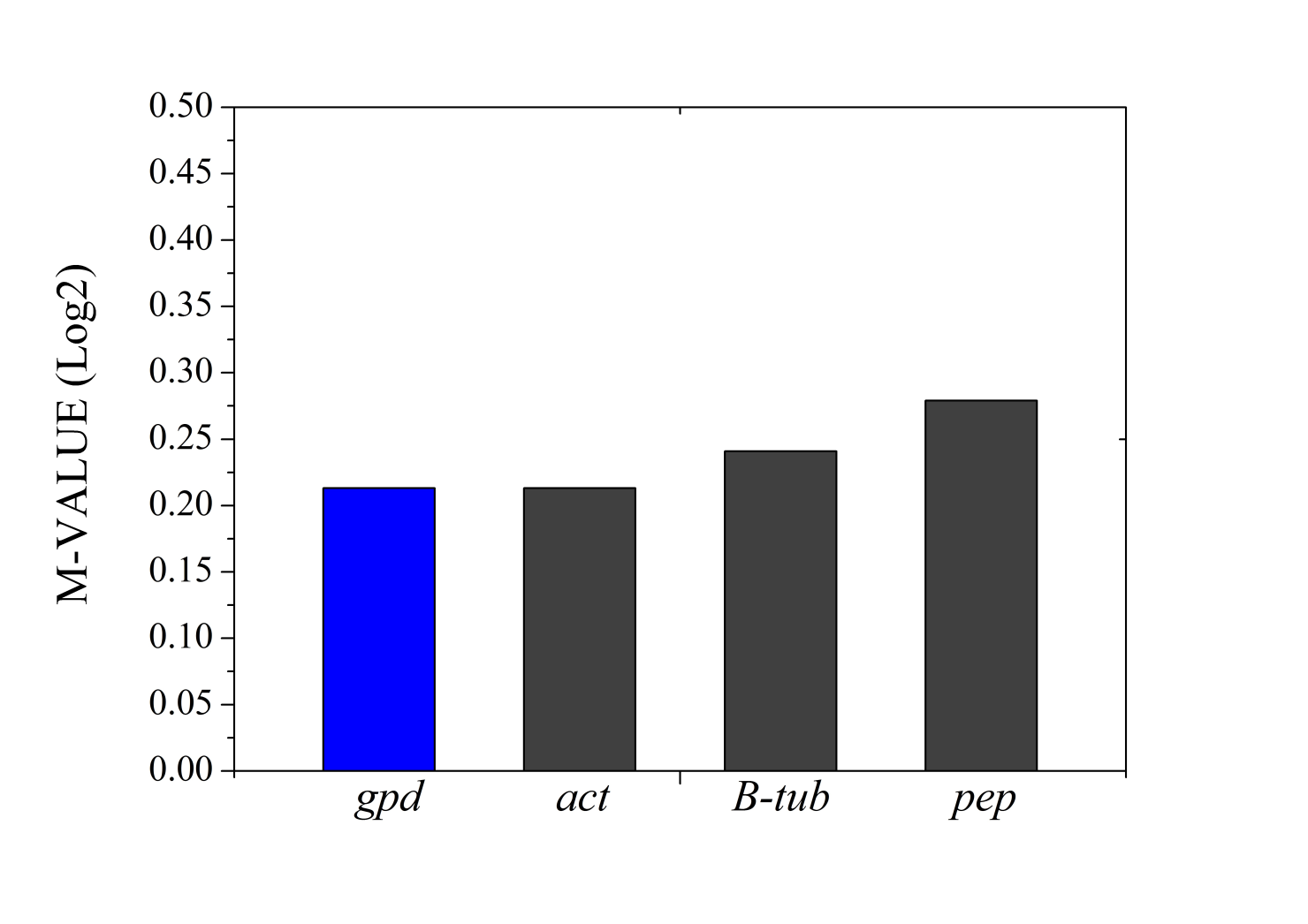


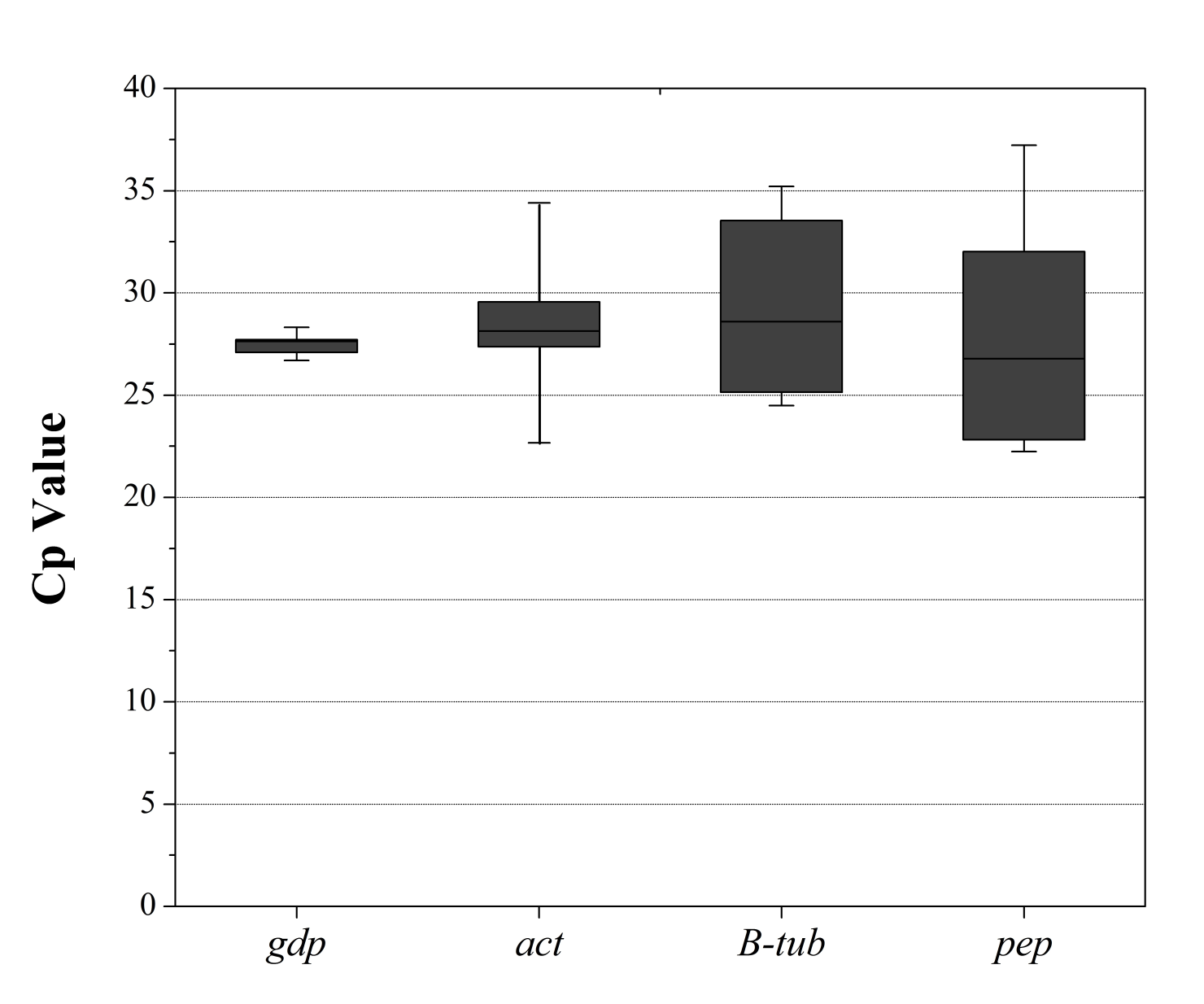

Supplement: Supplementary file 1 — 10.1186/s13568-016-0263-3 Growth of P. ostreatus and pH profile in submerged fermentations in BMF (●black circle), BBF (■ black square) and AYF (♦black diamond) media. The error bars represent the standard deviation of three different fermentation runs. Figure S2. Genorm analysis of the expression stability of 4 reference genes. Figure S3. Variability of Cp values of 4 reference genes tested under the 3 different fermentation conditions using NormFinder. Table S1. Identifiers and product lengths of reference genes primers used in this study. [file 13568_2016_263_MOESM1_ESM.docx]
